# A Machine Learning Model to Predict Intravenous Immunoglobulin-Resistant Kawasaki Disease Patients: A Retrospective Study Based on the Chongqing Population

**DOI:** 10.3389/fped.2021.756095

**Published:** 2021-11-08

**Authors:** Jie Liu, Jian Zhang, Haodong Huang, Yunting Wang, Zuyue Zhang, Yunfeng Ma, Xiangqian He

**Affiliations:** ^1^School of Medical Informatics, Chongqing Medical University, Chongqing, China; ^2^Medical Data Science Academy, Chongqing Medical University, Chongqing, China

**Keywords:** machine learning, Kawasaki disease, intravenous immunoglobulin resistance, risk factors, prediction model

## Abstract

**Objective:** We explored the risk factors for intravenous immunoglobulin (IVIG) resistance in children with Kawasaki disease (KD) and constructed a prediction model based on machine learning algorithms.

**Methods:** A retrospective study including 1,398 KD patients hospitalized in 7 affiliated hospitals of Chongqing Medical University from January 2015 to August 2020 was conducted. All patients were divided into IVIG-responsive and IVIG-resistant groups, which were randomly divided into training and validation sets. The independent risk factors were determined using logistic regression analysis. Logistic regression nomograms, support vector machine (SVM), XGBoost and LightGBM prediction models were constructed and compared with the previous models.

**Results:** In total, 1,240 out of 1,398 patients were IVIG responders, while 158 were resistant to IVIG. According to the results of logistic regression analysis of the training set, four independent risk factors were identified, including total bilirubin (TBIL) (OR = 1.115, 95% CI 1.067–1.165), procalcitonin (PCT) (OR = 1.511, 95% CI 1.270–1.798), alanine aminotransferase (ALT) (OR = 1.013, 95% CI 1.008–1.018) and platelet count (PLT) (OR = 0.998, 95% CI 0.996–1). Logistic regression nomogram, SVM, XGBoost, and LightGBM prediction models were constructed based on the above independent risk factors. The sensitivity was 0.617, 0.681, 0.638, and 0.702, the specificity was 0.712, 0.841, 0.967, and 0.903, and the area under curve (AUC) was 0.731, 0.814, 0.804, and 0.874, respectively. Among the prediction models, the LightGBM model displayed the best ability for comprehensive prediction, with an AUC of 0.874, which surpassed the previous classic models of Egami (AUC = 0.581), Kobayashi (AUC = 0.524), Sano (AUC = 0.519), Fu (AUC = 0.578), and Formosa (AUC = 0.575).

**Conclusion:** The machine learning LightGBM prediction model for IVIG-resistant KD patients was superior to previous models. Our findings may help to accomplish early identification of the risk of IVIG resistance and improve their outcomes.

## Introduction

Kawasaki disease (KD) is an acute vasculitis disease with bilateral conjunctival inflammation and atypical rash as the main clinical features. It mainly occurs in infants under 5 years of age (1). The main complication of KD patients is coronary artery lesions (CALs), which are the main reason for the increase in the incidence of acquired heart disease in children (2). Prompt treatment with high-dose (2 g/kg) intravenous immunoglobulin (IVIG) could significantly reduce the manifestations of KD and CALs. However, 10–20% of KD patients are resistant to IVIG (3, 4). After initial IVIG administration, recrudescent or persistent fever may occur, and further treatment is required at 48 h after the initial use of IVIG, such as the second administration of IVIG and corticosteroids (5). Therefore, it is of great significance to accurately detect IVIG-resistant KD patients and implement appropriate regimens early. In the past 10 years, many scholars have conducted a large number of studies on IVIG resistance. Egami (6), Kobayashi (7), and Sano (8) constructed three scoring systems based on the characteristics of the Japanese population. Fu (9) retrospectively analyzed 1,177 KD patients and established a prediction model for Beijing children. In 2015, Lin et al. collected data from 248 KD children and constructed the Taiwanese Formosa scoring system (10). Although the abovementioned scoring systems have performed well in their respective research populations, due to the existence of genetic susceptibility, the prediction performance of these systems in Chongqing city is not good (Table 1) (11, 12), which precludes wide application in the early prediction of IVIG resistance in Chongqing. It remains a challenge to develop a new prediction model with better predictive performance for children in Chongqing city, one of the largest cities in western China.

**Table 1:** Effectiveness and validation of previous prediction models in Chongqing city.

**Scoring systems**	**Study population**	**Predictive effectiveness in the literature**		**Validation effectiveness in Chongqing**			
				**Xiao**		**Ye**	
		**Sensitivity**	**Specificity**	**Sensitivity**	**Specificity**	**Sensitivity**	**Specificity**
Egami	Japan	0.780	0.760	0.346	0.806	0.364	0.857
Kobayashi	Japan	0.860	0.670	0.580	0.855	0.443	0.843
Sano	Japan	0.846	0.870	0.283	0.913	0.207	0.957
Fu	Beijing, China	0.541	0.712	0.519	0.660	0.457	0.750
Formosa	Taiwan, China	0.714	0.810	0.827	0.286		

In recent years, with the rapid development of machine learning algorithms and model interpretation methods, machine learning has been applied in many different fields and has shown great potential in assisting clinical diagnosis (13–15). This study retrospectively analyzed the clinical data of 1,398 KD patients on the medical big data platform of Chongqing Medical University from January 2015 to August 2020 and applied machine learning algorithms to the construction of IVIG-resistant prediction models for exploration. A more suitable prediction model for IVIG-resistant KD in the Chongqing area was developed.

## Materials and Methods

### Patients

The data come from the medical big data platform of Chongqing Medical University, which contains the electronic medical record data of 7 medical institutions affiliated with Chongqing Medical University. According to the inclusion and exclusion criteria, the inpatient electronic medical records data of 1,398 patients diagnosed with KD who received treatment on the platform from January 2015 to August 2020 were selected. Inclusion criteria: (1) Comply with the diagnostic criteria for KD in the diagnostic guidelines of Kawasaki Disease Version 5 (16); (2) Receive IVIG (2 g/kg) during the acute period treatment; (3) Complete clinical data. Exclusion criteria: (1) Incomplete KD and other confusing diseases, such as toddler's idiopathic arthritis; (2) Rehospitalized due to recurrence of KD; (3) Diagnosed with KD outside the hospital and receiving IVIG treatment; (4) Incomplete clinical data. This study was approved by the Ethics Committee of Chongqing Medical University.

### Definition and Data Collection

IVIG-resistant KD was defined as KD patients with a persistent or recurrence of fever ≥38°C at any time from 36 h to 2 weeks after initial IVIG treatment accompanied by one or more of the main symptoms (17).

The presence of coronary artery lesions was defined as coronary artery diameter ≥2.5 mm in patients aged 0–3 years old, ≥3.0 mm in patients aged 3–9 years old and ≥3.5 mm in patients aged older than 9 years (18).

All demographic characteristics, clinical features, imaging data and laboratory data prior to the initial use of IVIG were collected. The demographic characteristics included age (month) and sex; clinical features included days of illness at the initial treatment, maximum body temperature and cervical lymphadenopathy, conjunctival hyperemia, lip changes, rash, perianal changes, and edema of the hands and feet; imaging data prior to the initial use of IVIG included the presence of CALs. The laboratory data included blood cell analysis: neutrophil count, white blood cell (WBC), lymphocyte count, platelet count (PLT), hemoglobin (HB), percentage of neutrophils, neutrophil-to-lymphocyte ratio (NLR), platelet-to-lymphocyte ratio (PLR); biochemical examination: lactic dehydrogenase (LDH), total bilirubin (TBIL), globulin, albumin, alanine transaminase (ALT), gamma-glutamyl transpeptidase (GGT), aspartate aminotransferase (AST), procalcitonin (PCT), serum sodium, serum potassium, C-reactive protein (CRP), and erythrocyte sedimentation rate (ESR) (Table 2).

**Table 2:** Clinical information as features.

**Categories**	**Number of variables**	**Variables**
Basic information	2	Age in months^a^, ^b^, Sex
Clinical features	8	Days of illness at the initial treatment^a^, ^b^, ^d^, Maximum temperature, Cervical lymphadenopathy^e^, Conjunctival hyperemia, Lip changes, Rash^d^, Perianal changes^d^, edema of the hands and feet
Imaging data	1	Presence of CALs
Biochemical examination	12	LDH, TBIL^c^, Globulin, Albumin^e^, ALT^b^, GGT, AST^a^, ^c^, Serum potassium, Serum sodium^a^, CRP ^a^, ^b^, ^c^, ^d^, ESR, PCT
Blood cell analysis	8	Neutrophil count, WBC, Lymphocyte count, PLT ^a^, ^b^, HB, Percentage of neutrophils ^a^, ^d^, ^e^, NLR, PLR

a *Variables used in Kobayashi score*.

b *Variables used in Egami score*.

c *Variables used in Sano score*.

d *Variables used in Fu score*.

e *Variables used in Formosa score*.

### Statistical Analysis

Statistical analysis was performed by SPSS version 25.0. We used frequency (percentage) to describe categorical variables, and a χ^2^*-*test was used to analyze the difference between IVIG-responsive and IVIG-resistant groups. Since all continuous variables were non-normally distributed, the median (interquartile range) was used to present and compare results by the Mann-Whitney U test. *P* < 0.05 was considered statistically significant. The statistically significant variables in the univariate analysis were included in the logistic regression analysis to further screen out independent risk factors for IVIG resistance, and the selected risk factors were incorporated into the machine learning models to establish the IVIG-resistant KD prediction model. Sensitivity, specificity, and area under the curve (AUC) were used to evaluate the prediction performance of the models.

### Machine Learning Algorithm Prediction Model Construction

We used the computer-generated random number method to divide 1,398 KD patients into a training set (979 cases) and a test set (419 cases) at a ratio of 7:3. The training set was used for model training, and the test set was used to verify the generalization ability of the models. Using the “univariate analysis + logistic regression analysis” method to screen variables from the training set, we built a logistic regression nomogram, support vector machine (SVM), XGBoost, and LightGBM machine learning algorithm prediction models.

The rms package of R language (R version 3.6.3) was used to build a logistic regression nomogram.

The Scikit-learn package was adopted in the Python 3.6.5 environment to implement the SVM and XGBoost prediction models. SVM training process: the penalty term coefficient C was set to 0.5, the original dimension was expanded by a linear kernel function, and the hyperplane that maximizes the separation of the two groups in the high-dimensional space was found to obtain the best prediction model. XGBoost model training process: the learning rate was set to 0.2, and the depth of the tree was set to 3. The Python language LightGBM package was used to build the LightGBM prediction model. The learning rate was set to 0.02, and the maximum depth of the tree was 6 (The training process of model parameters has been uploaded in the form of attachment). Due to the severe imbalance between IVIG-responsive and IVIG-resistant groups, we used the class_weight parameter to adjust the weight of the positive and negative samples in the classifier (19) to increase the importance of a small number of sample categories and improve the classification performance of the model.

## Results

### Demographic Features

A total of 1,398 KD patients were included in this study. There were 158 cases in the IVIG-resistant group (accounting for 11.3%), including 64 female children (40.5%), 94 male (59.5%), and 28.5 months of onset (18, 44.25); 1,240 cases were in the IVIG-responsive group (88.7%), with 472 cases of female children (38.1%), 768 cases of male children (61.9%), and age of onset of 29 (17.00, 48.00).

### Analysis of Risk Factors for IVIG Resistance in the Training set

Among the 979 patients in the training set, 111 patients were in the IVIG-resistant group, and 868 patients were in the IVIG-responsive group. According to univariate analysis (Table 3), 14 variables were significantly different between the two groups (*P* < 0.05), including the days of illness at the initial treatment, lymphocyte count, PLT, HB, percentage of neutrophils, NLR, TBIL, globulin, albumin, ALT, GGT, AST, PCT, and serum sodium. There was no significant difference in the remaining 17 variables (*P* > 0.05).

**Table 3:** Univariate analysis comparison of clinical indexes in the training set.

**Variable**	**IVIG responsive**	**IVIG responsive**	***x^**2**^/Z***	***P***
	**(*n* = 111)**	**(*n* = 868)**		
**Basic information**				
Age in months (month)	30 (18–41)	29 (17–45)	−0.249	0.803
Sex (male)	64 (57.6%)	529 (60.9%)	0.445	0.505
**Clinical features**				
Days of illness at the initial treatment (day)	6 (5–7)	6 (5–7)	−2.670	0.008
Maximum temperature (°C)	39.8 (39.5–40.1)	39.7 (39.3–40.0)	−1.684	0.092
Cervical lymphadenopathy (positive)	91 (81.9%)	759 (87.4%)	2.565	0.109
Conjunctival hyperemia (positive)	90 (81%)	746 (85.9%)	1.866	0.172
Lip changes (positive)	107 (96.3%)	857 (98.7%)	3.561	0.059
Rash (positive)	70 (63.0%)	572 (65.8%)	0.351	0.554
Perianal changes (positive)	52 (46.8%)	333 (38.3%)	2.968	0.085
Edema of the hands and feet (positive)	78 (70.2%)	565 (65.0%)	1.171	0.279
**Laboratory examination**				
Neutrophil count (10^∧^9/L)	10.67 (7.35–12.71)	9.61 (7.10–11.70)	−1.758	0.079
WBC (10^∧^9/L)	14.39 (10.84–18.54)	14.69 (11.23–18.14)	−0.652	0.514
Lymphocyte count (10^∧^9/L)	3.11 (1.82–3.75)	3.36 (2.53–3.94)	−3.326	0.001
PLT (10^∧^9/L)	343.00 (271.00–403.00)	376.00 (295.00–469.00)	−3.657	<0.01
HB (g/dL)	103.00 (99.00–111.00)	107.00 (100.00–113.00)	−2.230	0.026
Percentage of neutrophils (%)	76.00 (63.00–83.00)	68.00 (54.00–79.00)	−4.209	<0.01
LDH (U/L)	258.40 (230.00–295.50)	268.50 (226.00–307.95)	−0.179	0.858
NLR	3.34 (2.23–5.54)	2.86 (1.94–4.21)	−3.762	<0.01
PLR	115.79 (82.09–179.72)	115.62 (84.29–160.25)	−0.625	0.532
TBIL (μmol/L)	8.6 (5.2–20)	6.70 (4.00–8.58)	−5.421	<0.01
Globulin (g/L)	21.4 (17.7–24.7)	22.5 (19.2–25.4)	−2.564	0.010
Albumin (g/L)	35.2 (31.7–38.4)	36.7 (33.7–39.6)	−3.476	0.001
ALT (U/L)	70.1 (21.8–127.3)	32.60 (17.15–46.15)	−5.887	<0.01
GGT (U/L)	81.7 (16.9–147.20)	35.25 (15.93–78.25)	−4.136	<0.01
AST (U/L)	36.5 (26–44.1)	29.35 (22.50–34.4)	−5.094	<0.01
PCT (μmol/L)	2.33 (0.49–4.71)	0.91 (0.25–1.81)	−6.517	<0.01
Serum potassium (mmol/L)	4.11 (3.72–4.45)	4.16 (3.81–4.54)	−1.801	0.072
Serum sodium (mmol/L)	136.4 (133.8–138.5)	137.3 (135.5–139.1)	−3.654	<0.01
CRP (mg/L)	49 (27–82)	45.00 (24.25–68.00)	−1.332	0.183
ESR (mm/h)	70 (49–85)	69.90 (48.25–90.00)	−0.273	0.785
**Imaging data**				
Presence of CALs (positive)	40 (36.0%)	289 (33.2%)	0.331	0.565

The 14 variables with statistical significance in the univariate analysis were used as independent variables, and the occurrence of IVIG resistance was used as the dependent variable (Yes = 1, No = 0). Logistic regression analysis was performed (α_in_ = 0.05, α_out_ = 0.10). The results showed that the four variables TBIL, PCT, ALT, and PLT were statistically significant (*P* < 0.05) and were independent risk factors for IVIG-resistant KD patients (Table 4).

**Table 4:** Logistic regression analysis in the training set.

**Risk factors**	***OR***	**95% CI**	***P***
Days of illness at the initial treatment	0.946	0.782–1.143	0.564
Serum sodium	0.965	0.884–1.054	0.431
Globulin	1.008	0.946–1.074	0.813
TBIL	1.115	1.067–1.165	<0.01
Albumin	0.971	0.915–1.03	0.328
PCT	1.511	1.270–1.798	<0.01
GGT	0.997	0.992–1.001	0.178
ALT	1.013	1.008–1.018	<0.01
AST	1.011	0.995–1.026	0.168
HB	0.986	0.961–1.011	0.271
PLT	0.998	0.996–1	0.028
Percentage of neutrophils	6.474	0.877–47.804	0.067
Lymphocyte count	1.129	0.905–1.408	0.283
NLR	0.98	0.906–1.06	0.609

### Logistic Regression Nomogram and Machine Learning Model Construction

**Figure 1:**
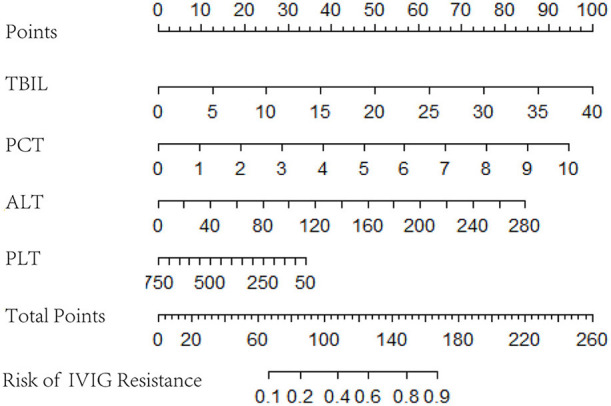
Logistic regression nomogram.

**Figure 2:**
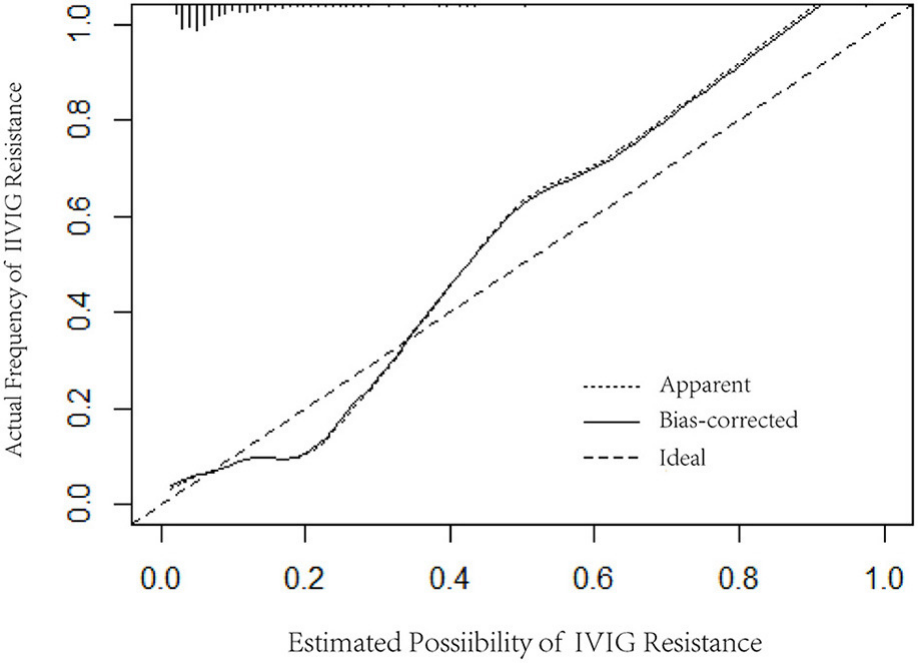
Consistency analysis of the logistic regression nomogram.

Logistic regression analysis results were used to screen variables, including TBIL, PCT, ALT, and PLT, to construct a logistic regression nomogram, SVM, XGBoost, and LightGBM machine learning prediction models. The results of the logistic regression nomogram prediction model are shown in Figure 1, and Figure 2 shows the consistency analysis of the logistic regression nomogram prediction model. The results show that the model has good stability.

**Figure 3:**
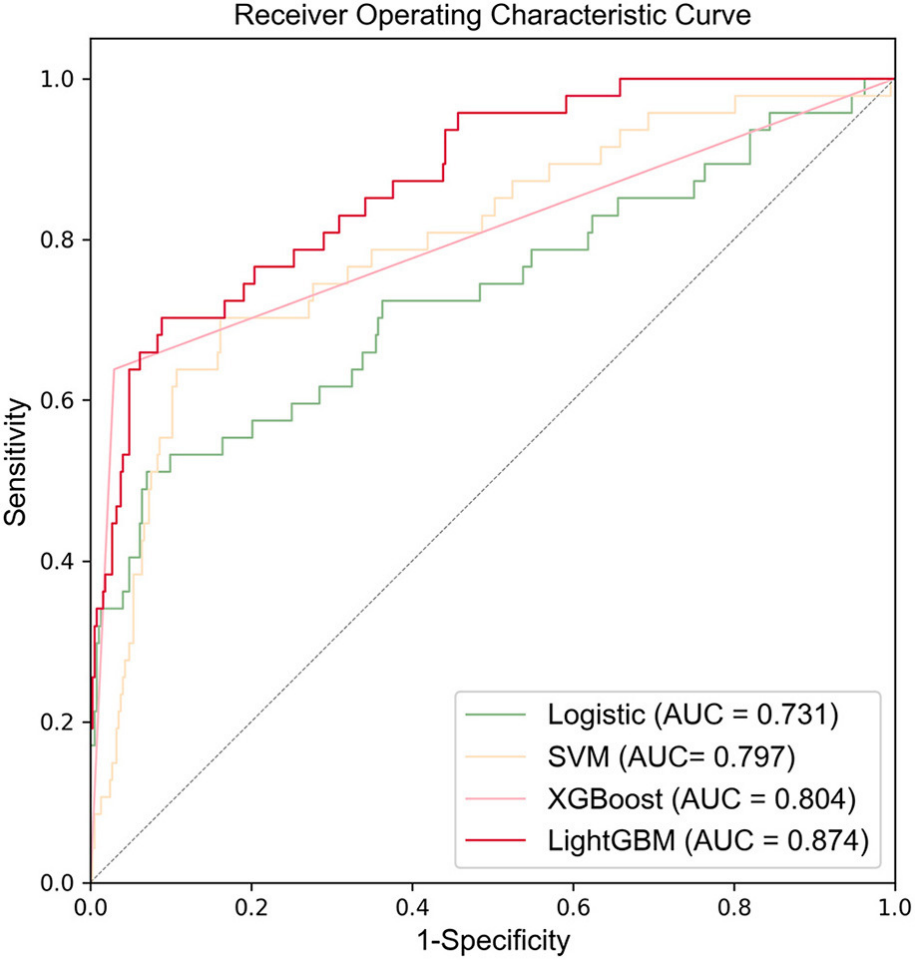
ROC curves of four models in the test set.

Figure 3 shows the ROC curves and AUC results of the four prediction models. Table 5 shows the specific evaluation indicators of the model. The LightGBM model had the highest sensitivity and AUC value, with 0.702 and 0.874, respectively. The model with the highest specificity was the XGBoost model (specificity = 0.967). Combining the three indicators of sensitivity, specificity, and AUC value, the LightGBM model achieved a significantly better predictive performance.

**Table 5:** Comparison of prediction efficiency of four models in the test set.

**Model**	**Sensitivity**	**Specificity**	**AUC**
Logistic	0.617	0.712	0.731
SVM	0.681	0.841	0.797
XGBoost	0.638	0.967	0.804
LightGBM	0.702	0.903	0.874

### Comparison With the Previous Scoring Systems

Compared with the previous IVIG-resistant scoring systems, the AUC value of the LightGBM model (AUC = 0.874) was higher than those of Egami (AUC = 0.581), Kobayashi (AUC = 0.524), Sano (AUC = 0.519), Fu (AUC = 0.578), and Formosa (AUC = 0.575). The Formosa scoring system had the highest sensitivity (sensitivity = 0.762) but low specificity (specificity = 0.393). The model with the highest specificity was the Egami scoring system (specificity = 0.931). With comprehensive sensitivity, specificity, and AUC values, the LightGBM model had the highest predictive performance (Table 6).

**Table 6:** Comparison between the new model and five other IVIG resistance KD prediction models.

**Model**	**Sensitivity**	**Specificity**	**AUC**
LightGBM	0.702	0.903	0.874
Egami	0.234	0.931	0.581
Kobayashi	0.134	0.911	0.524
Sano	0.272	0.773	0.519
Fu	0.234	0.841	0.578
Formosa	0.762	0.393	0.575

## Discussion

The main complication of KD is coronary artery lesions, which have gradually replaced rheumatic fever and become the main cause of childhood acquired heart disease. Currently, the treatment of KD mainly depends on high-dose IVIG; however, IVIG-resistant KD is not sensitive to IVIG, and additional treatment cannot quickly and effectively reduce vascular inflammation after the initial use of IVIG.

Therefore, there is an urgent need to build a prediction model for IVIG-resistant KD with high predictive ability for specific populations in Chongqing areas. Here, we reviewed 1,398 KD patients in 7 medical institutions affiliated with Chongqing Medical University. The logistic regression method with strong interpretability was used to screen variables, and four risk factors for IVIG resistance were screened out, including TBIL, PCT, ALT, and PLT. A variety of machine learning algorithms have been applied to build prediction models with high complexity. These models performed well in sensitivity, specificity and AUC and appear to be superior to previous models when applied to the Chongqing KD population.

In the past decade, logistic regression has been the first choice to build IVIG prediction models due to its simple model and strong interpretability. The Kobayashi score, Egami score, Formosa score and most other predictive scores were based on logistic regression. When the classification boundary is linear, the logistic regression model has a better prediction effect (20), but when processing high-latitude, large-volume data, the effect is often not good. With the development of artificial intelligence algorithms, an increasing number of machine learning algorithms have been developed, including traditional K-nearest neighbors, decision trees, SVM algorithms, and the emerging XGBoost and LightGBM models. These machine learning models offer excellent performance in processing high-latitude and large-volume data. An increasing number of scholars are applying machine learning algorithms to clinical research (21–23). In this study, logistic regression nomograms and SVM, XGBoost, and LightGBM algorithms were used to construct IVIG resistance prediction models. Among the constructed models, the LightGBM model had the best comprehensive predictive performance, with a sensitivity of 0.702, a specificity of 0.903, and an AUC value of 0.874. The LightGBM algorithm is a gradient boosting framework based on the decision tree algorithm released by Microsoft Research Asia in 2017. It uses the leafwise algorithm with depth restrictions and discards the levelwise algorithm used by XGBoost. More errors can be reduced with the same number of splits, so the LightGBM algorithm achieves better accuracy than other models (24).

In terms of variable screening, TBIL was included in our study as a high-risk factor for IVIG resistance. This is consistent with the findings of Sano et al. (8), who used total bilirubin ≥0.9 mg/dL as a predictor of IVIG resistance. The increase in TBIL in KD patients in the IVIG-resistant group may be related to acute hepatic vascular inflammation leading to hepatic vascular congestion and hepatic vascular inflammation leading to liver cell damage. Several large-scale cross-sectional studies have revealed a strong correlation between the presence of cardiovascular disease and the concentration of serum total bilirubin. Schwertner et al. (25) observed for the first time that there was a significant negative correlation between serum total bilirubin and the prevalence of coronary ischemic disease. Subsequently, experiments by Hopkins (26) and Breimer (27) successively confirmed this conclusion. Egami (6) hypothesizes that ALT≥80 IU/L is an important risk factor for IVIG resistance. Liu (28) also showed that KD patients with higher ALT levels are more likely to develop IVIG resistance. This is consistent with our research results. PCT is a common serum marker of inflammation, and it increases in severe bacterial infections. The latest research by some scholars has shown that PCT concentrations below 0.25 ng/ml may help distinguish KD from sepsis, and PCT concentrations of 0.25–0.50 ng/ml may help predict IVIG resistance (29). PLTs are the first responders to vascular injury and endothelial rupture, but studies have shown that PLTs are also inflammatory effector cells, with various activities ranging from acute inflammation to adaptive immunity (30). There are a large number of receptors on the surface of PLTs, and these receptors often interact with other cells (WBCs and endothelial cells). *In vitro* experiments showed that human neutrophils partially rely on platelets to enhance fibrin deposition in the bloodstream (31). In this study, thrombocytopenia in KD patients in the IVIG-resistant group may be related to the continuous consumption of platelets due to coronary artery lesions.

In summary, this study was based on the interpretability of logistic regression to screen independent risk variables and construct logistic regression nomogram, SVM, XGBoost, and LightGBM prediction models for IVIG resistance. The new models exhibited better prediction efficiency than the previous models and can be widely used in theory, but this study also presents some limitations. First, this study was a retrospective analysis, and the results need to be further verified by prospective studies. Second, some data items were missing, which might result in bias in the statistical analysis. In future studies, we will conduct prospective studies and collect more data to improve the screening of IVIG-resistant risk factors and model construction to further evaluate the effectiveness of new models.

## Data Availability Statement

The original contributions presented in the study are included in the article/Supplementary Material, further inquiries can be directed to the corresponding author.

## Author Contributions

JL and XH designed the study and analyzed the data. JL, JZ, HH, YW, ZZ, and YM acquired the data. JL and XH drafted the manuscript. JZ, HH, YW, ZZ, and YM read and revised the manuscript. All authors contributed to all study data, write and approved the final version of the manuscript.

## Funding

This study was supported by the Smart Medicine Research Project of Chongqing Medical University (Nos. ZHYX2019017 and YJSZHYX202015), the Humanities and Social Science Foundation of Chongqing Medical University (No. 201724), and the Innovation experiment project of School of medical information, Chongqing Medical University (No. 2019C010). All the financial expenditure of this study comes from the above programs.

## Conflict of Interest

The authors declare that the research was conducted in the absence of any commercial or financial relationships that could be construed as a potential conflict of interest.

## Publisher's Note

All claims expressed in this article are solely those of the authors and do not necessarily represent those of their affiliated organizations, or those of the publisher, the editors and the reviewers. Any product that may be evaluated in this article, or claim that may be made by its manufacturer, is not guaranteed or endorsed by the publisher.

## References

[1] Newburger JW Takahashi M Burns JC. Kawasaki disease. J Am Coll Cardiol. (2016) 67:1738–49. 10.1016/j.jacc.2015.12.073 27056781

[2] Kobayashi T Ayusawa M Suzuki H Abe J Ito S Kato T. Revision of diagnostic guidelines for Kawasaki disease (sixth revised edition). Pediatr Int. (2020) 62:1135–8. 10.1111/ped.14326 33001522

[3] Sleeper LA Minich LL McCrindle BM Li JS Mason W Colan SD. Evaluation of Kawasaki disease risk-scoring systems for intravenous immunoglobulin resistance. J Pediatr. (2011) 158: 831–835.e3. 10.1016/j.jpeds.2010.10.031 21168857 PMC3075321

[4] Lee SM Lee JB Go YB Song HY Lee BJ Kwak JH. Prediction of resistance to standard intravenous immunoglobulin therapy in Kawasaki disease. Korean Circ J. (2014) 44:415–22. 10.4070/kcj.2014.44.6.415 25469144 PMC4248614

[5] Brogan PA Bose A Burgner D Shingadia D Tulloh R Michie C. Kawasaki disease: an evidence based approach to diagnosis, treatment, and proposals for future research. Arch Dis Child. (2002) 86:286–90. 10.1136/adc.86.4.286 11919108 PMC1719139

[6] Egami K Muta H Ishii M Suda K Sugahara Y Iemura M. Prediction of resistance to intravenous immunoglobulin treatment in patients with Kawasaki disease. J Pediatr. (2006) 149:237–40. 10.1016/j.jpeds.2006.03.050 16887442

[7] Kobayashi T Inoue Y Takeuchi K Okada Y Tamura K Tomomasa T. Prediction of intravenous immunoglobulin unresponsiveness in patients with Kawasaki disease. Circulation. (2006) 113:2606–12. 10.1161/CIRCULATIONAHA.105.592865 16735679

[8] Sano T Kurotobi S Matsuzaki K Yamamoto T Maki I Miki K. Prediction of non-responsiveness to standard high-dose gamma-globulin therapy in patients with acute Kawasaki disease before starting initial treatment. Eur J Pediatr. (2007) 166:131–7. 10.1007/s00431-006-0223-z 16896641

[9] Fu PP Du ZD Pan YS. Novel predictors of intravenous immunoglobulin resistance in Chinese children with Kawasaki disease. Pediatr Infect Dis J. (2013) 32:e319–23. 10.1097/INF.0b013e31828e887f 23446442

[10] Lin MT Chang CH Sun LC Liu HM Chang HW Chen CA. Risk factors and derived formosa score for intravenous immunoglobulin unresponsiveness in Taiwanese children with Kawasaki disease. J Formos Med Assoc. (2016) 115:350–5. 10.1016/j.jfma.2015.03.012 25910931

[11] Xiao L Zhang J Yi L Qiu L Yang Y Ye XC. Predictive analysis of intravenous immunoglobulin unresponsive Kawasaki disease. J Clin Pediatrics. (2018) 36:765 (in Chinese) . 10.3969/j.issn.1000-3606.2018.10.01031269967

[12] Ye XC Zhang J. Evaluation of the efficiency of different scoring systems in predicting intravenous immunoglobulin unresponsiveness in Kawasaki disease. Chin. J. Evid. Based Pediatrics. (2016) 11:337–340. (in Chinese). 10.3969/j.issn.1673-5501.2016.05.004

[13] Le Berre C Sandborn WJ Aridhi S Devignes MD Fournier L Smaïl-Tabbone M. Application of artificial intelligence to gastroenterology and hepatology. Gastroenterology. (2020) 158:76–94.e2. 10.1053/j.gastro.2019.08.058 31593701

[14] Sato M Morimoto K Kajihara S Tateishi R Shiina S Koike K. Machine-learning approach for the development of a novel predictive model for the diagnosis of hepatocellular carcinoma. Sci Rep. (2019) 9:7704. 10.1038/s41598-019-44022-8 31147560 PMC6543030

[15] Heo J Yoon JG Park H Kim YD Nam HS Heo JH. Machine learning-based model for prediction of outcomes in acute stroke. Stroke. (2019) 50:1263–5. 10.1161/STROKEAHA.118.024293 30890116

[16] Ayusawa M Sonobe T Uemura S Ogawa S Nakamura Y Kiyosawa N. Revision of diagnostic guidelines for Kawasaki disease (the 5th revised edition). Pediatr Int. (2005) 47:232–4. 10.1111/j.1442-200x.2005.02033.x 15771703

[17] McCrindle BW Rowley AH Newburger JW Burns JC Bolger AF Gewitz M. Diagnosis, treatment, and long-term management of kawasaki disease: a scientific statement for health professionals from the American Heart Association. Circulation. (2017) 135:e927–99. 10.1161/CIR.0000000000000484 28356445

[18] Wang Y Wang W Gong F Fu S Zhang Q Hu J. Evaluation of intravenous immunoglobulin resistance and coronary artery lesions in relation to Th1/Th2 cytokine profiles in patients with Kawasaki disease. Arthritis Rheum. (2013) 65:805–14. 10.1002/art.37815 23440694

[19] Ting KM. An instance-weighting method to induce cost-sensitive trees. IEEE Trans Knowl Data En. (2002) 14:659–65. 10.1109/TKDE.2002.1000348

[20] Takeuchi M Inuzuka R Hayashi T Shindo T Hirata Y Shimizu N. Novel risk assessment tool for immunoglobulin resistance in Kawasaki disease: application using a random forest classifier. Pediatr Infect Dis J. (2017) 36:821–6. 10.1097/INF.0000000000001621 28441265

[21] Ramroach S Joshi A John M. Optimisation of cancer classification by machine learning generates an enriched list of candidate drug targets and biomarkers. Mol Omics. (2020) 16:113–25. 10.1039/c9mo00198k 32095794

[22] Chiu CC Lee KT Lee HH Wang JJ Sun DP Huang CC. Comparison of models for predicting quality of life after surgical resection of hepatocellular carcinoma: a prospective study. J Gastrointest Surg. (2018) 22:1724–31. 10.1007/s11605-018-3833-7 29916106

[23] Mišić VV Gabel E Hofer I Rajaram K Mahajan A. Machine learning prediction of postoperative emergency department hospital readmission. Anesthesiology. (2020) 132:968–80. 10.1097/ALN.0000000000003140 32011336

[24] Hu HT Wang Z Kuang M Wang W. Need for normalization: the non-standard reference standard for microvascular invasion diagnosis in hepatocellular carcinoma. World J Surg Oncol. (2018) 16:50. 10.1186/s12957-018-1347-0 29514674 PMC5842608

[25] Schwertner HA Jackson WG Tolan G. Association of low serum concentration of bilirubin with increased risk of coronary artery disease. Clin Chem. (1994) 40:18–23. 8287538

[26] Hopkins PN Wu LL Hunt SC James BC Vincent GM Williams RR. Higher serum bilirubin is associated with decreased risk for early familial coronary artery disease. Arterioscler Thromb Vasc Biol. (1996) 16:250–5. 10.1161/01.atv.16.2.250 8620339

[27] Breimer LH Wannamethee G Ebrahim S Shaper AG. Serum bilirubin and risk of ischemic heart disease in middle-aged British men. Clin Chem. (1995) 41:1504–8. 7586525

[28] Liu G Wang S Du Z. Risk factors of intravenous immunoglobulin resistance in children with kawasaki disease: a meta-analysis of case-control studies. Front Pediatr. (2020) 8:187. 10.3389/fped.2020.00187 32373568 PMC7186309

[29] Niu MM Jiang Q Ruan JW Liu HH Chen WX Qiu Z. Clinical implications of procalcitonin in Kawasaki disease: a useful candidate for differentiating from sepsis and evaluating IVIG responsiveness. Clin Exp Med. (2021) 2021:1–11. 10.1007/s10238-021-00709-9 33839960 PMC8036161

[30] Semple JW Italiano JJ Freedman J. Platelets and the immune continuum. Nat Rev Immunol. (2011) 11:264–74. 10.1038/nri2956 21436837

[31] Barnard MR Linden MD Frelinger AR Li Y Fox ML Furman MI Michelson AD. Effects of platelet binding on whole blood flow cytometry assays of monocyte and neutrophil procoagulant activity. J Thromb Haemost. (2005) 3:2563–70. 10.1111/j.1538-7836.2005.01603.x 16241954

