# Supplementary material for: A Machine Learning Model to Predict Intravenous Immunoglobulin-Resistant Kawasaki Disease Patients: A Retrospective Study Based on the Chongqing Population
Source: Front Pediatr. 2021 Nov 8;9:756095. doi: 10.3389/fped.2021.756095 (PMC8606736; doi:10.3389/fped.2021.756095)
Supplement: Supplementary file 2 [file Data_Sheet_2.pdf]

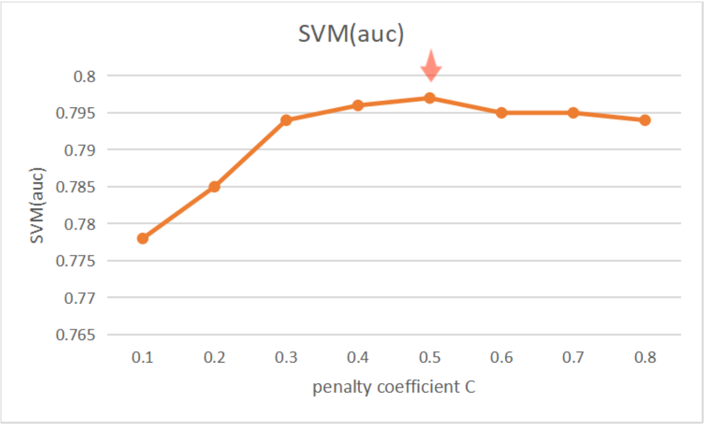

Figure 1 SVM parameter training process

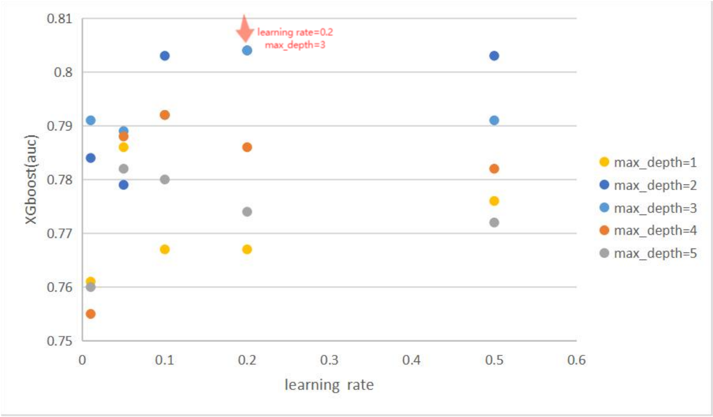

Figure 2 XGBoost parameter training process

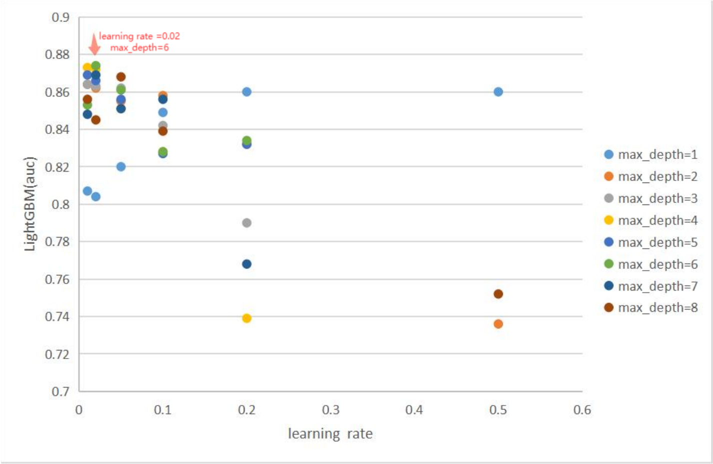

Figure 3 LightGBM parameter training process
